# Dermoscopy Use Leads to Earlier Cutaneous Melanoma Diagnosis in Terms of Invasiveness and Size? A Single-Center, Retrospective Experience

**DOI:** 10.3390/jcm11164912

**Published:** 2022-08-21

**Authors:** Gianluca Nazzaro, Emanuela Passoni, Fabio Pozzessere, Carlo Alberto Maronese, Angelo Valerio Marzano

**Affiliations:** 1 Dermatology Unit, Fondazione IRCCS Ca’ Granda Ospedale Maggiore Policlinico, Via Pace, 9, 20122 Milan, Italy; 2 Department of Pathophysiology and Transplantation, Università degli Studi di Milano, 20122 Milan, Italy

**Keywords:** dermoscopy, nevus, melanoma, skin oncology

## Abstract

Background: The incidence of cutaneous melanoma has risen in recent years. The aim of this study was to compare cutaneous melanomas diagnosed at the Dermatology Unit of Fondazione IRCCS Ca’ Granda Ospedale Maggiore Policlinico, Milan, Italy, from 2006 to 2020 and between two specific biennia, i.e., 2006–2007 and 2019–2020. Methods: Retrospective chart review, with dermoscopic image collection, of cutaneous melanomas diagnosed at the Fondazione IRCCS Ca’ Granda Ospedale Maggiore Policlinico, Milan, Italy, from 1 January 2006 to 31 December 2020 Results: A statistically significant increase was shown in the proportions of in situ melanoma and melanoma measuring less than 6 mm, i.e., small-diameter melanoma (SDM), across the studied period (*p* < 0.001). Moreover, in the biennium 2006–2007, among 220 melanoma diagnoses, 6 were in situ (2.7%), as compared with 68 melanomas in situ out of a total of 236 (28.8%) melanomas diagnosed in the biennium 2019–2020. A statistically significant difference in the proportion of in situ melanoma between the two biennia was demonstrated (*p* < 0.001). Furthermore, during the first biennium, 27/220 (12.3%) SDM were identified, as compared with 61/236 (25.9%) in the last. A statistically significant difference was shown in the proportion of SDM between the two (*p* < 0.001). Conclusions: The percentage of in situ melanomas and those that can be detected at a diameter <6 mm has increased. The latter has been shown to be around one-third of excised lesions, thus undermining the practicality of the ABCD mnemonic. Dermoscopic criteria for SDM are needed to help further refine melanoma diagnosis.

## 1. Introduction

The incidence of cutaneous melanoma has risen significantly in recent years. The reasons behind this trend are incompletely understood, although overdiagnosis is likely to have a role [1].

In parallel, dermoscopy has become more and more predominant in daily practice, with progressive improvements and equipment refinements [2,3,4,5] Indeed, after adequate training [6], dermoscopy has proved more accurate than clinical examination for the diagnosis of melanoma in the setting of pigmented skin lesions [7], improving the malignant/benign ratio in excised lesions [8]. It can be argued that the wider and wider adoption of dermoscopy, along with other possible factors at play (such as increased patient awareness and screening campaigns), could have had a measurable effect over the years on the characteristics of melanoma at the time of diagnosis, allowing its detection at an earlier stage. The aim of this research was to collect and compare cutaneous melanoma diagnoses rendered at the Dermatology Unit of the Fondazione IRCCS Ca’ Granda Ospedale Maggiore Policlinico, Milan, Italy from 1 January 2006 to 31 December 2020. Further, we focused our comparison on two biennia, i.e., 2006–2007 and 2019–2020, respectively the first and the last in the range at our disposal.

## 2. Materials and Methods

The dermopathology archive of the Fondazione IRCCS Ca’ Granda Ospedale Maggiore Policlinico, Milan, Italy, was searched for histological diagnoses of malignant melanoma (MM) from 1 January 2006 to 31 December 2020.

The following variables were collected: age at the time of the diagnosis, gender, Breslow thickness, and maximum longitudinal diameter, as measured on dermoscopy.

Melanomas were classified into two categories, i.e., melanoma in situ (MIS) and invasive melanoma. Lesions were dichotomized also based on their dermoscopic diameter. Melanomas with a diameter < 6 mm were defined as small-diameter melanomas (SDM).

Diagnoses rendered in the period from 1 January 2006 to 31 December 2007 (group A) and those made from 1 January 2019 to 31 December 2020 (group B) were chosen for comparison.

Results were expressed as counts and percentages or as medians and interquartile range (IQR).

Trend analysis across the studied period (2006–2020) was carried out using the general linear model for continuous variables (melanoma counts, median age at diagnosis) and the Mantel–Haenzsel test for trend for proportions (sex, MIS, SDM), respectively.

A Chi-square test was performed in order to assess the difference in the proportion of MIS and SDM between the two groups.

*p*-values below 0.05, two-sided, were considered statistically significant (IBM SPSS Statistics for Windows, version 27.0, IBM Corp., Armonk, NY, USA).

## 3. Results

**Figure 1:**
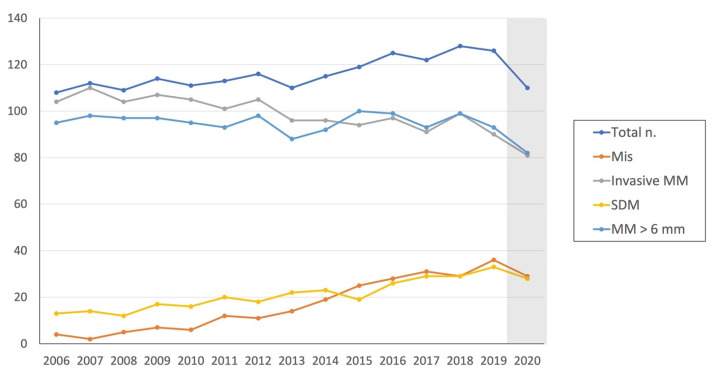
Trends in melanoma diagnoses from 1 January 2006 to 31 December 2020 at the Fondazione IRCCS Ca’ Granda Ospedale Maggiore Policlinico, Milan, Italy. The gray column in 2020 symbolizes the beginning of the era of COVID-19.

Trends in melanoma diagnoses from 1 January 2006 to 31 December 2020 at the Dermatology Unit of Fondazione IRCCS Ca’ Granda Ospedale Maggiore Policlinico, Milan, Italy, are illustrated in Figure 1. Demographics, counts of MIS, and invasive melanomas, as well as SDM and those >6 mm, are summarized in Table 1.

A statistically significant increase was noted in the total number of melanomas diagnosed between 1 January 2006 and 31 December 2020. No trend for age (*p* = 0.41) or sex (*p* = 0.34) was documented across time.

A statistically significant increase was documented for the proportions of MIS (test for trend, *p* < 0.001) and SDM (test for trend, *p* < 0.001) in the same period. The annual mean percent change was +1.62% for MIS and +0.96% for SDM.

These trends retained statistical significance after adjusting for age and sex (*p* < 0.001).

Further, in the period from 1 January 2006 to 31 December 2007 (group A), 220 melanomas were diagnosed, while, in the period from 1 January 2019 to 31 December 2020 (group B), 236 diagnoses of melanoma were collected.

There were 118 (53.6%) and 114 (48.3%) male patients in the first and second group, respectively.

Median age at the time of the diagnosis was 55 years (IQR: 41.75–69) in the first biennium and 63 years (IQR: 51–73) in the second one.

Six MIS were diagnosed during the first biennium (2006–2007) (2.7% of group A), while 68 MIS were recorded in the second one (2019–2020) (28.8% of group B). A statistically significant difference in the percentage of MIS diagnoses between the two groups was demonstrated (*p* < 0.001), meaning that melanomas excised in the 2019–2020 biennium were more likely to be in situ compared to melanomas excised in the 2006–2007 biennium.

Twenty-seven SDMs were diagnosed during the first biennium (12.3% of group A), whereas 61 SDMs were recorded during the second one (25.9% of group B). A statistically significant difference was shown in the percentage of SDMs between the two groups (*p* < 0.001), meaning that melanomas excised in the 2019–2020 biennium were more likely to be <6 mm diameter compared to lesions excised in the 2006–2007 biennium (Table 2).

Unsurprisingly, when assessing melanoma diagnoses rendered in 2020, the year of the COVID-19 pandemic, a decrease in the total number of melanomas diagnosed (−12.7%) was documented relative to 2019 (Figure 1). However, the proportion of cases diagnosed with invasive MM was approximately the same: 90/126 (71.4%) in 2019 vs. 81/110 (73.6%) in 2020.

## 4. Discussion

The findings of our single-center investigation show an incidence increase in MIS over the last fifteen years, this being in line with previous evidence from European and American studies [9,10,11,12,13]. Although we cannot confidently attribute this trend to dermoscopy exclusively and recognize the possibility that other factors may be at play (e.g., increased patient awareness), the addition of methodical dermoscopic evaluation represented the single, most evident change in routine clinical practice across the two biennia in our view.

Indeed, the entire dermatopathology unit and most of the dermatology unit remained the same over the years. It is also unlikely that behavioral changes in the population can account for such a dramatic change over just 15 years.

The reduction in the number of melanoma diagnoses in 2020 (as compared with 2019) was minor and likely related to the COVID-19 pandemic. However, reports concerning the impact of COVID-19 in the number of annual melanoma diagnoses have been inconsistent [14].

In the last decades, the incidence of cutaneous melanoma rose considerably in Caucasians, with percentage annual increases ranging from 3% to 7% [9]. The annual percentage increase in MIS incidence was greater than that of invasive MM, averaging a 14% incidence increase every year versus an average 4.5% incidence increase every year for invasive MM [10]. Indeed, such an increase in melanoma diagnoses seems to be mainly driven by MIS. Moreover, the slight incidence increase of invasive MM appears to be related mainly to thin neoplasms [11]. The incidence of thick invasive MM has also increased over time, although with a slower rate.

This scenario, known as the “melanoma epidemic”, has led to controversies over the years. Two main possible explanations have been proposed. The first one supports the idea that we are witnessing a true incidence increase for cutaneous melanoma, not just an increase in the diagnostic ability to detect it. This is believed to be due to population behavioral changes in lifestyle associated with an increased exposure to UV radiation during the past decades, starting from the 1950s [15] However, studies investigating the difference in melanoma incidence in patients respectively under and over the age of 50 years old showed stabilization or even a slight decrease among the former. One may argue that this is the result of primary prevention and even more recent lifestyle changes, such as: reduced sun exposure in younger individuals, appropriate sun protection use, less outdoor work, and reduced outdoor leisure activities [15]. As already mentioned in the introduction, the second possible explanation for the incidence increase of cutaneous melanoma is overdiagnosis. This could be due to greater patient awareness and healthcare seeking behavior for skin cancer screening [15], as well as the increased capability of dermatologists to diagnose lesions at an earlier stage, especially thanks to the ever more frequent use of dermoscopy [2,3] over the last decades. Indeed, dermoscopy allows the diagnosis of cutaneous melanoma at an earlier stage, possibly determining the different incidence increases we are witnessing when comparing MIS and invasive MM. It is also possible that the adoption of ancillary techniques such as in vivo confocal microscopy (which, however, is not available at our institution) may have contributed to this trend, determining the excision of melanomas in a very early stage (i.e., earlier than what would have been done without it) [16].

Indeed, a decreasing trend in median tumor thickness over the years (2003–2017) was recently recorded in a registry-based study involving a significant proportion of the Italian population. This trend was paralleled by an increase in 5-year net survival rates, which, however, was only in part attributable to the former, according to the authors [17].

**Figure 2:**
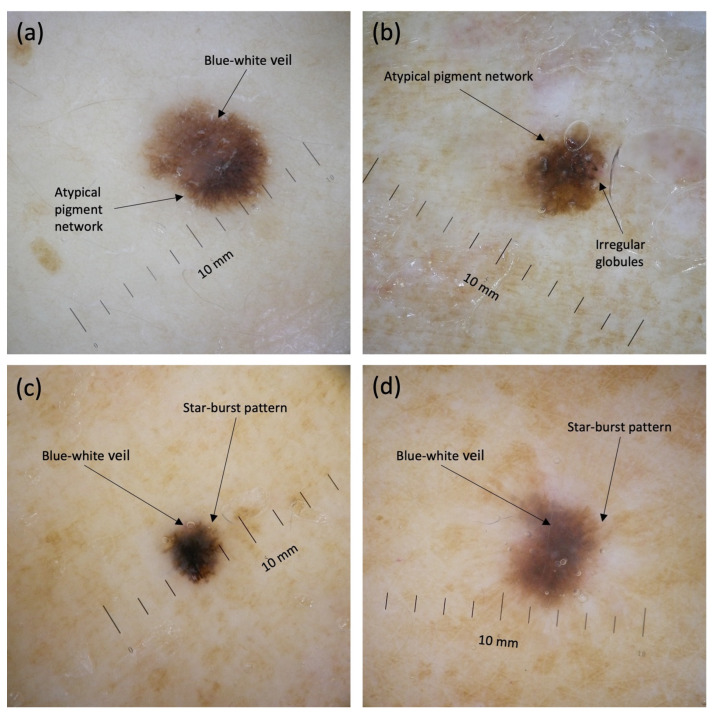
Dermoscopy of four representative small-diameter melanomas, including invasive pT1a (**a**,**c**,**d**) and in situ melanomas (**b**). Dermoscopic features of depicted lesions include an atypical pigment network (**a**,**b**), subtle irregular globules (**b**), a blue–white veil (**a**,**c**,**d**), and a starburst pattern (**c**,**d**).

Dermoscopy has been demonstrated to be more accurate than naked eye examination for the diagnosis of cutaneous melanoma when performed on suspicious skin lesions [18,19], while also allowing the accurate examination of smaller lesions. This, along with greater patient awareness both at an individual- and at the population-level, has likely led to an increase in the percentage of melanomas that are detected at a diameter < 6 mm (Figure 2), in our view. In the present study, the percentage of SDM has been shown to be around one third of excised lesions, thus undermining the practicality of the ABCD (asymmetry, border, color, diameter) mnemonic, which focuses heavily on lesions with a diameter > 6 mm [20]. Incidence trends and dermoscopic clues to detect SDMs have not been extensively investigated in the literature. Developing novel, accurate dermoscopic criteria for SDMs and/or adapting existing ones to this challenge could improve our ability to detect melanoma at an even earlier stage, countering the present risk of overdiagnosis.

## Figures and Tables

**Table 1:** Characteristics of cutaneous melanomas excised from 1 January 2006 to 31 December 2020 at the Dermatology Unit of Fondazione IRCCS Ca’ Granda Ospedale Maggiore Policlinico, Milan, Italy.

	Males	Median Age, Years (IQR)	MIS	Invasive MM	SDM (<0.6 mm)	MM ≥ 0.6 mm
**2006**	44 (40.74%)	54 (42–68.75)	4/108 (3.7%)	104/108	13/108 (12.04%)	95/108
**2007**	74 (66.07%)	55.5 (41–69)	2/112 (1.79%)	110/112	14/112 (12.50%)	98/112
**2008**	61 (55.96%)	55 (40.5–68)	5/109 (4.59%)	104/109	12/109 (11.01%)	97/109
**2009**	66 (57.89%)	54 (41–67.5)	7/114 (6.14%)	107/114	17/114 (14.91%)	97/114
**2010**	49 (44.14%)	60 (46.5–72)	6/111 (5.41%)	105/111	16/111 (14.41%)	95/111
**2011**	53 (46.90%)	53 (41.5–69)	12/113 (10.62%)	101/113	20/113 (17.70%)	93/113
**2012**	55 (47.41%)	56.5 (42–73.5)	11/116 (9.48%)	105/116	18/116 (15.52%)	98/116
**2013**	49 (44.55%)	61 (53.5–72.5)	14/110 (12.73%)	96/110	22/110 (20.00%)	88/110
**2014**	61 (53.04%)	59 (44–69.5)	19/115 (16.52%)	96/115	23/115 (20.00%)	92/115
**2015**	57 (47.90%)	58 (49–68.5)	25/119 (21.01%)	94/119	19/119 (15.97%)	100/119
**2016**	72 (57.60%)	62 (53.5–71)	28/125 (22.40%)	97/125	26/125 (20.80%)	99/125
**2017**	64 (52.46%)	56 (46.5–67)	31/122 (25.41%)	91/122	29/122 (23.77%)	93/122
**2018**	59 (46.09%)	59 (42–74.5)	29/128 (22.66%)	99/128	29/128 (22.66%)	99/128
**2019**	56 (44.44%)	63 (52–73)	36/126 (28.57%)	90/126	33/126 (26.19%)	93/126
**2020**	58 (52.73%)	63 (50–72)	29/110 (26.36%)	81/110	28/110 (25.45%)	82/110

MIS: Melanoma in situ; MM: malignant melanoma; SDM: small-diameter melanoma.

**Table 2:** Characteristics of cutaneous melanomas excised in the period 2006–2007 and 2019–2020 at the Dermatology Unit of Fondazione IRCCS Ca’ Granda Ospedale Maggiore Policlinico, Milan, Italy.

	Males	Median Age, Years (IQR)	MIS	Invasive MM	SDM (<0.6 mm)	MM ≥ 0.6 mm
**2006–2007 (Group A)**	118 (53.6%)	55 (41.75–69)	6/220 (2.7%)	214/220	27/220 (12.3%)	193/220
**2019–2020** **(Group B)**	114 (48.3%)	63 (51–73)	68/236 (28.8%)	168/236	61/236 (25.9%)	175/236

MIS: Melanoma in situ; MM: malignant melanoma; SDM: small-diameter melanoma.

## Data Availability

Data sharing is not applicable to this article, as no new data were created or analyzed in this study.
